# Extract of Pimento as a Revulsive

**Published:** 1878-04

**Authors:** 


Extract of Pimento as a Revulsive.—The extract of pimento has a beautiful red color, identical with that of the dried fruit. Suitably incorporated in a plastic mass, and spread upon squares of paper, its application is very easy. It is unnecessary to warm it, for it adheres sufficiently to the skin; but it is well, on parts subject to movement, to fix it with a bandage just as a blister. Moreover, its action may be augmented or moderated according to the pressure. On removal, the heat and tingling may be immediately arrested by the application of a little starch.—*Clinic.*

